# Depressive Symptoms and Healthcare Utilization in Late Life. Longitudinal Evidence From the AgeMooDe Study

**DOI:** 10.3389/fmed.2022.924309

**Published:** 2022-07-22

**Authors:** Elżbieta W. Buczak-Stec, Margrit Löbner, Janine Stein, Anne Stark, Hanna Kaduszkiewicz, Jochen Werle, Kathrin Heser, Birgitt Wiese, Siegfried Weyerer, Michael Wagner, Martin Scherer, Steffi G. Riedel-Heller, Hans-Helmut König, André Hajek

**Affiliations:** ^1^Department of Health Economics and Health Services Research, Hamburg Center for Health Economics, University Medical Center Hamburg-Eppendorf, Hamburg, Germany; ^2^Institute of Social Medicine, Occupational Health and Public Health, University of Leipzig, Leipzig, Germany; ^3^Department of General Practice and Primary Care, Center for Psychosocial Medicine, University Medical Center Hamburg-Eppendorf, Hamburg, Germany; ^4^Institute of General Practice, Medical Faculty, University of Kiel, Kiel, Germany; ^5^Central Institute of Mental Health, Medical Faculty Mannheim/Heidelberg University, Mannheim, Germany; ^6^Department of Neurodegenerative Diseases and Geriatric Psychiatry, University Hospital Bonn, Bonn, Germany; ^7^Institute of General Practice, Hannover Medical School, Hannover, Germany; ^8^Department of Psychiatry and Psychotherapy, University Hospital Bonn, Bonn, Germany

**Keywords:** depression, depressive symptoms, health care use, GP visits, hospitalization, specialist visit, health care utilization, Andersen model

## Abstract

**Objective:**

The aim of this study was to investigate the longitudinal impact of depressive symptoms on utilization of healthcare in terms of GP visits as well as specialist visits and hospital admission in late life among community-dwelling individuals.

**Methods:**

Longitudinal data (baseline and follow-up) were derived from the German multicentre, prospective cohort study “Late-life depression in primary care: needs, health care utilization and costs” study (AgeMooDe). At baseline, *n* = 1,230 patients aged 75 years and older were recruited from primary care practices. Main outcomes of interest were use of health care services: the number of GP visits, the number of medical specialist visits, and hospital admission. We used the Geriatric Depression Scale (GDS-15) to measure depression. Outcomes were analyzed with multilevel random intercept negative binominal regression and logistic random-effects models.

**Results:**

At baseline (*n* = 1,191), mean age was 80.7 (SD 4.6) years, 62.9% were female, and 196 individuals (16.5%) had depression (GDS-15 ≥6). Our longitudinal analyses indicated that older individuals with more depressive symptoms visited their GP more often (IRR=1.03; CI [1.01-1.04], *p* < 0.001), were visiting medical specialists more frequently (IRR=1.03; CI [1.01-1.04], *p* < 0.01), and had higher odds of being hospitalized (OR=1.08; CI [1.02-1.13], *p* < 0.01).

**Conclusions:**

Based on this large longitudinal study we showed that, after adjustment for important covariates, older individuals with more depressive symptoms had higher health care utilization over time. They visited their GP and specialists more frequently and they had higher odds of being hospitalized. This may suggest that higher utilization of specialist care and increased likelihood of being hospitalized may be also attributable to unspecific symptoms or symptoms that are elevated through depressive symptoms.

## Introduction

The prevalence of depression is high among older individuals (1, 2), representing one of the major concerns not only for geriatricians but also for general practitioners (GPs) and medical specialists. It is estimated that up to 13% of older individuals have a minor depression and between 6% and more than 40% have depressive symptoms (3). With progressive population aging, it is most likely that depressive symptoms in older age will be a widespread condition that should be properly acknowledged e.g., by addressing this issue by the physician. Depressive symptoms in older age are frequently associated with numerous adverse outcomes, among others, with cognitive impairment, worse clinical outcomes like higher in-hospital mortality, higher rates of readmission and also poorer quality of life (4–8). Moreover, the excess costs of depression are considerable, which can be attributed to both high medical and societal costs (9).

In recent years, great effort has been made, on one hand, to provide appropriate treatment for individuals with mental health problems and, on the other hand to reduce hurdles to seeking help (10). Depression may manifest differently in older individuals than in younger individuals. Therefore, the responsiveness to the mental health treatment options and health services utilization, not only related to mental health, may vary. Furthermore, potential gender differences in the association between depressive symptoms and health care use may be more pronounced in older age. As the prevalence of depressive symptoms differs among women and men (11) health care use may also differ with regard to sex.

In recent years, the impact of depressive symptoms on health care utilization among older individuals has been investigated by various studies (12–15). With regard to inpatient care, some studies have shown a positive association between depressive symptoms and hospital admissions as well as length of hospital stay (13, 16, 17). However, after considering important risk factors like comorbidities and functioning, this association often disappeared (18–20).

Further, the evidence concerning the association between depression and outpatient care, such as number of visits to the GP and to specialists, is more homogeneous (15, 21–25). Most studies have reported that patients with more depressive symptoms also have more GP visits (15, 21–24), mainly due to the fact that depressed individuals may have more unspecific symptoms which may cause an increased number of consultations (20).

Most of the existing studies are based on cross-sectional data and many do not control for important confounders. Furthermore, many studies did not include other healthcare sectors.

Consequently, little is known about the longitudinal role of depressive symptoms on health care utilization across healthcare sectors in *older age*. Therefore, the aim of this study was to investigate the longitudinal impact of depressive symptoms on utilization of healthcare in terms of GP visits as well as specialist visits and hospital admission in late life among community-dwelling individuals. Moreover, we examined whether this association varies with regard to sex.

## Materials and Methods

### Study Design and Participants

For this study, we used baseline and follow-up (FU) data from the German “Late-life depression in primary care: needs, health care utilization and costs” study (AgeMooDe). AgeMooDe is a longitudinal, multicentre, prospective cohort study that collected data from four centers in Germany (Bonn, Hamburg, Leipzig, and Mannheim).

Study participants were recruited from GP practices. Eligible patients were 75 years or older at baseline and had at minimum one contact with their GP within the last 6 months. At baseline, patients with severe illnesses that could be fatal within 3 months, with moderate and severe dementia, and patients with insufficient German language skills or unable to give consent were excluded. GPs and practice staff screened all eligible patients for depressive symptoms. For each patient diagnosed with depression, another eligible patient without depression was randomly selected. A detailed description of the recruitment process is provided by Stein et al. (26). Trained staff conducted a standardized clinical interview in patients' homes. The baseline data were collected from May 2012 to December 2013. At baseline, *n* = 1,230 of 1,356 recruited patients have participated. The follow-up took place ~1 year later (between May 2013 and December 2014, *n* = 982).

At baseline, a total of *n* = 1,191 patients provided responses on healthcare utilization and depressive symptoms (Geriatric Depression Scale GDS scores). At FU, these data were available for *n* = 965 patients. The main reasons for non-participation were refusal (62%), death (14%) and severe physical diseases or cognitive impairment (12%). More details are reported by Hoell et al. (27).

### Outcome Variables

Main outcomes of interest were the use of health care services: (i) the number of GP visits, (ii) the number of medical specialist visits (sum score of all specialist visits e.g., cardiologist, orthopaedist, dermatologist, pulmonologist), and (iii) hospital admission (dichotomized, yes or no). Health care use and the number of visits were self-reported by the patients using the Questionnaire for Health-Related Resource Use in Older Population (FIMA) (28). The FIMA is a generic questionnaire designed for an older population. Patients were asked whether they had visited the respective physician and used a specific service in the last 6 months.

### Independent Variables

#### Depressive Symptoms

Depressive symptoms were assessed with the validated 15-item Geriatric Depression Scale (GDS) (29). Respondents were asked a sequence of questions (binary response: yes; no) which referred to depressive symptoms (e.g., dropping of many activities, afraid something bad is going to happen, feeling worthless etc.). The sum score ranged from 0 to 15, with higher scores denoting more severe depressive symptoms. A threshold of six or more points indicated depression (30). This threshold is recommended for the German adaptation of the GDS-15 (31). The cut-off value of six demonstrated the best sensitivity and specificity (31).

#### Covariates

The inclusion of the relevant covariates in the analysis was based on previous research on health care utilization among older individuals (32). Moreover, we segregated factors into predisposing characteristics, enabling resources and somatic need factors, building on Andersen's model of health services use (33).

We included sociodemographic characteristics of the patients *(predisposing characteristics)–*age, sex, marital status (married living together; married living apart; single; divorced; widowed), and educational level [categorized according to CASMIN–in low, middle, high level (34)]. We also included household situation grouped into private household with others or spouse; private household alone; living in nursing or retirement home. Additionally, it was adjusted for the type of health care insurance (private and statutory health insurance) and social support (*enabling resources)*. Social support was assessed by the German Version of the ENRICHD Social Support Inventory–ESSI (35). It ranges from 5 to 25, with higher scores indicating higher levels of perceived social support. Scale values lower or equal to 18 indicated low social support (36). We also adjusted for a set of health status variables (*need factors)*. Functional impairment was assessed with a 24-item scale assessing (instrumental) activities of daily living (IADL/ADL). Patients who had difficulty on at least one ADL/IADL were classified as functionally impaired (37). The chronic disease score (CDS) was used to control for burden of multimorbidity (38). The CDS is based on pharmacy data and encompasses 17 weighted conditions (for some conditions e.g., respiratory illness, heart disease, weights depend on number of medications used). The score ranges from 0 to 12, with higher scores indicating higher morbidity. Cognitive functioning was assessed using the Mini-Mental State Examination (MMSE), with a total score ranging from 0 (worst) to 30 (best) (39). Additionally, we controlled for visual and hearing impairment. Patients were asked “Do you have visual and/or hearing difficulties”. Answers were dichotomized respectively (no impairment; mild, or severe impairment).

### Statistical Analysis

The bivariate associations were tested using *t*-tests and Pearson's χ^2^, as appropriate. The association between depressive symptoms and utilization of health care services (number of GP and medical visits) was analyzed with multilevel random intercept negative binominal regression. Incidence-rate ratios (IRR) with 95% confidence intervals (95% CI) are reported. Moreover, we used logistic random-effects model to analyse the association between depressive symptoms and hospital admission (40). Logistic random-effects models are appropriate for clustered dichotomous responses (40). Results are presented as the conditional odds ratios with 95% CI. In sensitivity analyses, it was tested whether the associations were robust to the used measurement namely continuous variable (depressive symptoms) or dichotomized variable (depression). All analyses were performed using Stata 16.0 (Stata-Corp, College Station, Texas, USA). The criterion for statistical significance was set at *p* < 0.05.

## Results

### Study Design and Participants

At baseline (*n* = 1,191), mean age was 80.7 (SD 4.6) years, 62.9% were female, and 196 individuals (16.5%) had depression (according to the GDS cut-off score of 6 and more points; GDS ≥6). The average number of GP visits within the last 6 months was 4.3 (SD 4.2), and the average number of specialist visits was 4.6 (SD 4.8), and 15.0% of the participants were hospitalized within the last 6 months.

In comparison to individuals without depression, individuals with depression were older, had less social support, had more chronic conditions, were more often functionally impaired, and had more often hearing and vision impairment (Table 1). Furthermore, patients with depression had a higher utilization of outpatient care (average number of GP visits 5.6 (SD 6.1) vs. 4.1 (SD 3.7), *p* < 0.001; average number of visits to medical specialists 5.4 (SD 5.0) vs. 4.5 (SD 4.8), *p* < 0.05)–and of inpatient care (hospital admissions 24.0 vs. 13.2%, *p* < 0.001) (Table 1).

**Table 1:** Descriptive characteristics of study cohort at baseline—total sample, depressive (GDS ≥6) and non-depressed individuals.

**Mean (SD); Range/*N* (%)**	**Total sample (*N* = 1,191)**	**No Depression (*N* = 995)**	**Depression (*N* = 196)**	***p*–value**
Age	80.73 (4.58); (75–98)	80.57 (4.48); (75–98)	81.53 (4.96); (75–94)	<0.01
Sex				<0.05
Female	749 (62.9%)	610 (61.3%)	139 (70.9%)	
Male	442 (37.1%)	385 (38.7%)	57 (29.1%)	
Education (CASMIN)				0.198
Low	651 (54.9%)	537 (54.3%)	114 (58.2%)	
Middle	317 (26.8%)	262 (26.5%)	55 (28.1%)	
High	217 (18.3%)	190 (19.2%)	27 (13.8%)	
Marital status				<0.001
Married, living together with spouse	544 (45.7%)	481 (48.4%)	63 (32.1%)	
Married, living separated with spouse	24 (2.0%)	18 (1.8%)	6 (3.1%)	
Single	53 (4.5%)	48 (4.8%)	5 (2.6%)	
Divorced	72 (6.1%)	50 (5.0%)	22 (11.2%)	
Widowed	497 (41.8%)	397 (39.9%)	100 (51.0%)	
Living situation				<0.001
Alone (private household)	517 (43.6%)	415 (41.8%)	102 (52.3%)	
Living together with spouse/ partner/relative	602 (50.7%)	530 (53.4%)	72 (36.9%)	
Retirement home / assisted living / nursing home	68 (5.7%)	47 (4.7%)	21 (10.8%)	
Health Insurance				0.748
Private health insurance	54 (4.6%)	46 (4.7%)	8 (4.1%)	
Statutory health insurance	1,129 (95.4%)	943 (95.3%)	186 (95.9%)	
Social Support (ESSI)				<0.001
Low	220 (18.9%)	162 (16.6%)	58 (31.0%)	
High	942 (81.1%)	813 (83.4%)	129 (69.0%)	
Cognitive functioning (MMSE)	27.25 (2.46); (13–30)	27.41 (2.29); (13–30)	26.37 (3.08); (14–30)	<0.001
Chronic disease score (CDS)	5.11 (2.87); (0–12)	4.98 (2.86); (0–12)	5.85 (2.83); (0–12)	<0.001
Functional impairment (ADL)				<0.001
No	439 (36.9%)	406 (40.8%)	33 (16.8%)	
Yes	752 (63.1%)	589 (59.2%)	163 (83.2%)	
Visual impairment				<0.001
No	831 (69.8%)	739 (74.3%)	92 (46.9%)	
Yes	360 (30.2%)	256 (25.7%)	104 (53.1%)	
Hearing impairment				<0.01
No	675 (56.7%)	582 (58.5%)	93 (47.4%)	
Yes	516 (43.3%)	413 (41.5%)	103 (52.6%)	
*Outcome variables:*				
General practitioner visits	4.33 (4.21); (0–60)	4.08 (3.68); (0–48)	5.63 (6.07); (0–60)	<0.001
Specialist visits	4.63 (4.84); (0–76)	4.49 (4.80); (0–76)	5.37 (5.00); (0–33)	<0.05
Hospital visit				<0.001
No	1,013 (85.1%)	864 (86.8%)	149 (76.0%)	
Yes	178 (14.9%)	131 (13.2%)	47 (24.0%)	

*AgeMooDe Study—“Late-life depression in primary care: needs, health care utilization and costs”*.

*SD, standard deviation; p-values are based on chi-square or Fisher exact tests for categorical variables and on independent t tests for continuous variables; Depression—short version of the Geriatric Depression Scale, which contains 15 items (GDS-SF) (29); Educational level (CASMIN) low, middle, high [33]; Social support—German Version of the ENRICHD Social Support Inventory—ESSI (35), range 5-25, values lower or equal to 18 indicated low social support (36); Cognitive function—Mini-Mental State Examination (MMSE), from 0 (worst) to 30 (best) (39); Chronic disease score (CDS)—burden of multimorbidity, range 0–12, higher scores indicate higher morbidity (38); Functional impairment-−24-item scale (instrumental) activities of daily living (IADL/ADL). Patients who had difficulty on at least one ADL/IADL were classified as functionally impaired (37)*.

### Regression Analysis

#### Health Care Utilization and Depressive Symptoms

Associations between depressive symptoms and health care utilization are shown in Table 2. Results of the regression analysis showed that after controlling for predisposing, enabling and somatic need factors, depressive symptoms were consistently positively associated with all outcomes studied. Our longitudinal analyses indicated that older individuals with more depressive symptoms visited their GP more often (IRR = 1.03; CI [1.01–1.04], *p* < 0.001), were visiting medical specialists more frequently (IRR = 1.03; CI [1.01–1.04], *p* < 0.01), and had higher odds of being hospitalized (OR = 1.08; CI [1.02–1.13], *p* < 0.01).

**Table 2:** Determinants of health care utilization (GP visits, specialist visits and hospital admission) in late life among community-dwelling individuals.

	**Number of GP visits**		**Medical specialists–number of visits**		**Hospital admission (yes/no)**	
**Independent variables**	**IRR**	**95%CI**	**IRR**	**95%CI**	**OR**	**95%CI**
Depressive symptoms (GDS)	1.03***	(1.01–1.04)	1.03**	(1.01–1.04)	1.08**	(1.02–1.13)
Age	1.01	(1.00–1.02)	0.97***	(0.96–0.98)	0.98	(0.95–1.01)
Female (ref: male)	1.10*	(1.00–1.20)	0.99	(0.89–1.11)	0.71*	(0.51–0.98)
Education (CASMIN) (ref: low)						
Middle	0.97	(0.89–1.06)	1.08	(0.93–1.20)	0.85	(0.56–1.22)
High	0.99	(0.89–1.11)	1.06	(0.97–1.68)	0.83	(0.66–3.72)
Marital status: (ref: married, living with spouse)						
Married, living separated	0.98	(0.76–1.26)	1.28+	(0.97–1.68)	1.57	(0.66–3.72)
Single	0.91	(0.73–1.13)	0.97	(0.75–1.25)	1.00	(0.44–2.26)
Divorced	1.30**	(1.07–1.58)	1.06	(0.83–1.35)	1.58	(0.78–3.20)
Widowed	1.06	(0.92–1.22)	1.08	(0.91–1.28)	0.96	(0.56–1.67)
Living situation: (ref: alone)						
Living together with spouse/partner/relative	1.03	(0.90–1.18)	1.14	(0.97–1.34)	0.72	(0.43–1.23)
Retirement home/assisted living/ nursing home	1.10	(0.93–1.29)	0.98	(0.80–1.20)	0.86	(0.47–1.58)
Health insurance: (ref: statutory)						
Private health insurance	1.37***	(1.14–1.64)	0.83	(0.65–1.05)	1.64	(0.88–3.06)
High Social support (ESSI) (ref: low)	1.04	(0.95–1.14)	1.11*	(1.00–1.24)	1.17	(0.81–1.69)
Cognitive function (MMSE)	0.98*	(0.97–1.00)	1.00	(0.98–1.02)	1.02	(0.96–1.08)
Chronic disease score (CDS)	1.05***	(1.03–1.06)	1.02**	(1.01–1.04)	1.11***	(1.06–1.17)
Functional impairment (ADL) (ref: no)	1.16***	(1.07–1.25)	1.08+	(0.99–1.17)	1.74**	(1.25–2.42)
Visual impairment (ref: no)	0.92*	(0.85–1.00)	1.01	(0.92–1.10)	1.06	(0.79–1.44)
Hearing impairment (ref: no)	0.97	(0.90–1.05)	1.14**	(1.04–1.24)	1.26	(0.95–1.67)
Constant	8.25***	(3.26–20.86)	56.77***	(18.44–174.78)	0.39	(0.02–10.24)
Random part	Yes		Yes		Yes	
Observations	1,843		1,846		1,843	
Individuals	1,118		1,119		1,118	

*Results of multilevel random intercept negative binominal regression (IRR) and the random-effects logistic regression (OR). AgeMooDe–German “Late-life depression in primary care: needs, health care utilization and costs”*.

Incidence-rate ratios (IRR) and odds-ratios (OR) with 95% confidence intervals (95% CI) are reported. Depression—short version of the Geriatric Depression Scale, which contains 15 items (GDS-SF) (29); Educational level (CASMIN) low, middle, high [33]; Social support—German Version of the ENRICHD Social Support Inventory—ESSI (35), range 5–25, values lower or equal to 18 indicated low social support (36); Cognitive function–Mini-Mental State Examination (MMSE), from 0 (worst) to 30 (best) (39); Chronic disease score (CDS)—burden of multimorbidity, range 0–12, higher scores indicate higher morbidity (38); Functional impairment-−24-item scale (instrumental) activities of daily living (IADL/ADL). Patients who had difficulty on at least one ADL/IADL were classified as functionally impaired (37). ***p <0.001, **p <0.01, *p <0.05.

#### Correlates of Number of GP Visits

In addition to depressive symptoms, results revealed that women visited GPs more frequently than men (IRR=1.10; CI [1.00–1.20], *p* < 0.05). Moreover, the number of GP visits was positively associated with more chronic conditions (IRR=1.05; CI [1.03–1.06], *p* < 0.001) and with functional impairment (IRR=1.16; CI [1.07–1.25], *p* < 0.001). Individuals with better cognitive functioning were visiting GP less often (IRR=0.98; CI [0.97–1.00], *p* < 0.05). Furthermore, individual that had full private insurance (ref. statutory health insurance) visited GPs more often (IRR = 1.37; CI [1.14–1.64]; *p* < 0.001).

#### Correlates of Number of Medical Specialist Visits

Results of the regression analyses showed that apart from depressive symptoms, a higher number of specialist visits was associated with more chronic conditions (IRR = 1.02; CI [1.01–1.04], *p* < 0.001) and higher social support (IRR = 1.11; CI [1.00–1.24], *p* < 0.05). Moreover, older individuals visited medical specialists less frequently than younger individuals (IRR = 0.97; CI [0.96–0.98], *p* < 0.05). The number of specialist visits was not associated with predisposing variables such as sex, education, and enabling variable–insurance status. Furthermore, it was also not associated with cognitive functioning.

#### Correlates of Hospital Admissions

With regard to hospital admission, the analysis showed that, apart from depressive symptoms, individuals with functional impairment (OR = 1.74; CI [1.25–2.42], *p* < 0.01), and those with more chronic conditions (OR = 1.11; CI [1.06–1.17], *p* < 0.001) had higher odds of being hospitalized. Women had lower odds of being hospitalized than men (OR = 0.71; CI [0.51–0.98], *p* < 0.05). Education, health care insurance status and cognitive impairment were not significantly associated with hospital admission.

### Sensitivity Analysis

In sensitivity analysis depressive symptoms were measured by dichotomized variable (depression; no depression). The main results remained the same, namely depression was positively associated with health care utilization in every health care sector. Individuals with depression visited their GP more often (IRR = 1.19; CI [1.08–1.31], *p* < 0.001), were visiting medical specialists more frequently (IRR = 1.12; CI [1.00–1.26], *p* < 0.05), and had higher odds of being hospitalized (OR = 1.62; CI [1.15–2.29], *p* < 0.01). It was also examined whether sex moderates the association between depression and the outcomes. However, the interaction effects did not achieve statistical significance in all models. Additionally, we conducted regressions stratified by sex. Results showed that depressive symptoms were associated with all three outcomes in both sexes (visiting medical specialists and hospital admission was significant at *p* < 0.10 level). Please see Supplementary Tables S1, S2.

## Discussion

In this large longitudinal study of older adults, we demonstrated that, after adjustment for important covariates, older adults with more depressive symptoms had higher health care utilization. Older individuals with more depressive symptoms visited their GP and specialists more frequently. Moreover, they had higher odds of being admitted to the hospital. Our study extends current knowledge by showing that this association exists in every healthcare sector studied, namely in primary care, in outpatient specialist care and in inpatient care. Furthermore, our study adds to the existing evidence, mainly based on cross-sectional data, by showing that this relationship also exists longitudinally.

Our findings extends recent studies from several countries which showed a positive association between depressive symptoms and health care utilization among older individuals (12, 13, 19). Several possible explanations may provide an insight into this association. First, the increased use of health care services may be particularly attributable to the elevated use of mental health care services. In case of our analysis, this may apply to GP services, as GPs in Germany also provide certain mental health services. Our findings may also suggest that the patients with mental disorders may not have sufficient access to psychiatric health services or did not want to be treated by the psychiatrist and therefore refer to GPs for help (41, 42). However, future research in this area is required. This finding also indicates that the importance of good clinical training of GPs regarding the diagnosis of depression. The increased use of specialists services and higher likelihood of hospitalization among individuals with depressive symptoms, may be primary caused by other factors. First, commonly, individuals with depressive symptoms may have more (psycho-) somatic complaints (1, 3). Additionally, they could have an increased risk of developing further conditions or aggravate their existing conditions (triggered by depressive symptoms) (1), which may lead to increased healthcare utilization across those health care sectors in the long-term. This elevated health care use could possibly be further attributed to medication non-adherence and non-adherence to the medical treatment (23, 43). However, upcoming studies are needed to test these assumptions.

In terms of need variables, our study showed that factors such as higher number of chronic diseases and presence of functional impairment were consistently associated with higher health care utilization. Our findings are in line with previous research stating that need factors heavily drive doctor visits (44, 45).

Furthermore, our study underlines the importance of enabling factors with regard to health care utilization. Noteworthy, associations with health care use have been observed with regard to type of health insurance and social support in our study. We showed that privately insured individuals have a higher number of GP visits compared to those with statutory insurance. Our results suggest that the type of the health insurance has an important role in primary care (GPs), but not in the utilization of medical specialists and hospitalisations. This may suggest that in second-level treatment (outpatient specialist care and inpatient care), the need for medical care is of greater relevance than the insurance type old aged individuals are covered by. The importance of health insurance coverage for physician visits, but not for e.g., emergency room visits, has been demonstrated in other studies (13). However, more research in this area is needed. Furthermore, our results revealed that individuals with more social support were visiting medical specialists more frequently. A possible explanation may be that older individuals with better social support are encouraged by family and close friends to visit medical specialists.

Our analysis revealed that most of the predisposing characteristics such as education or marital status were not associated with health care utilization. However, we observed some important gender differences. After adjusting for covariates, women were more likely than men to visit GPs, whereas men had higher odds of being admitted to the hospital. Though, gender did not moderate the association between depressive symptoms and healthcare use. Overall, the evidence for gender differences in health care utilization is inconclusive (12, 23, 24). Some studies had shown that significant gender differences exists (46) e.g., in a large, nationally representative study, it has been found that women (65 years and older) were significantly more likely to utilize outpatient care then men (15). On the other hand, Lacruz et al. showed that that gender differences disappears once the comorbidities are controlled for (23). Numerous other studies have also found no significant gender differences [e.g., (13, 24, 47)]. The synthesis of the evidence for gender effect is challenging since the results vary, among others, on how the depressive symptoms were quantified (e.g., ICD-codes, GDS (Geriatric Depression Scale), CES-D (Center for Epidemiologic Studies Depression Scale), PHQ (Patient Health Questionnaire) control variables included in regression analysis, and the population included).

One possible explanation for observed gender differences (46, 48) is that older women may seek help promptly to the occurrence of any symptoms, whereas older men may tend to avoid or postpone doctor visits–which may lead to more serious conditions over time that result in hospital admission. A second possible explanation is, when older men decide to consult a physician, even for minor conditions, they may prefer to be treated by the specialists in the hospital rather than the family doctor. Such as gender-specific gaps in seeking care were shown in previous studies (46, 49–51). For example, it has been shown that in case of mental health problem, women typically seek care by theirs GPs more often than men (49). Gender differences in health care utilization and the underlying reasons for these differences should be investigated in future research.

### Strengths and Limitations

A major strength of our study is the use of data from a large multicentre longitudinal study. We considered health care utilization across the three main health care sectors, namely GPs visits, medical specialist visits and hospital admission. We used validated and well-established measures. In addition, depressive symptoms were measured both at baseline and at follow-up. Our study also has some limitations. First, health care utilization was inquired retrospectively which may cause some recall bias. However, it has been shown that there is a substantial correlation between data that are based on self-reported information and electronic medical records (52). Furthermore, patients did not provide information on a specific reason for hospital admission. Moreover, it is most likely that individuals with severe cognitive impairment did not participate in this study. Second, as the study was conducted in large cities in Germany, the generalizability to rural areas is difficult. While, in our study, depression was measured using a widely used short version form of GDS, in psychiatric research other tools to quantify depression are also common (such as interviews). GDS-15 is a validated measure with very good psychometric properties (31).

## Conclusions

Based on large longitudinal study we showed that, after adjustment for important covariates, older individuals with more depressive symptoms had higher health care utilization over time. They visited GPs and specialists more frequently and they had higher odds of being hospitalized. This may suggest that higher utilization of specialist care and increased likelihood of being hospitalized may be also attributable to unspecific symptoms or symptoms that are elevated through depressive symptoms.

## Data Availability Statement

Due to ethical restrictions involving patient data, underlying data are available upon request from the Working Group Medical Statistics and IT-Infrastructure. Contact information: BW, wiese.birgitt@mh-hannover.de.

## Ethics Statement

The Ethics Committees of all participating centers approved of the study (Ethics approval Leipzig: 020-12-23012012). The patients/participants provided their written informed consent to participate in this study.

## Author Contributions

EB-S made substantial contributions to the conception and design of the study, the analysis and interpretation of data, and drafted the manuscript. AH made substantial contributions to the interpretation of data and drafting of the manuscript and was responsible for supervision. ML, JS, AS, HK, JW, KH, BW, SW, MW, MS, SR-H, and H-HK made substantial contributions. All authors contributed to the article and approved the submitted version.

## Funding

This study was funded by the German Federal Ministry of Education and Research (BMBF), Grant number: (01GY1155A) and the Federal Ministry of Health (BMG), Grant number: (II A 5-2513 FSB 014).

## Conflict of Interest

The authors declare that the research was conducted in the absence of any commercial or financial relationships that could be construed as a potential conflict of interest.

## Publisher's Note

All claims expressed in this article are solely those of the authors and do not necessarily represent those of their affiliated organizations, or those of the publisher, the editors and the reviewers. Any product that may be evaluated in this article, or claim that may be made by its manufacturer, is not guaranteed or endorsed by the publisher.
