# Supplementary material for: Depressive Symptoms and Healthcare Utilization in Late Life. Longitudinal Evidence From the AgeMooDe Study
Source: Front Med (Lausanne). 2022 Jul 22;9:924309. doi: 10.3389/fmed.2022.924309 (PMC9354619; doi:10.3389/fmed.2022.924309)
Supplement: Supplementary file 1 [file Table_1.pdf]

Supplementary data

Table S1

Table S2

Table S1 Determinants of health care utilisation (GP visits, specialist visits and hospital admission) in late life among community-dwelling individuals. Results of multilevel random intercept negative binominal regression (IRR) and the random-effects logistic regression (OR). AgeMooDe - German "Late-life depression in primary care: needs, health care utilisation and costs".

	Number of GP visits		Medical specialists – number of visits		Hospital admission (yes/no)	
Independent variables	IRR	95%CI	IRR	95%CI	OR	95%CI
Depression (GDS ≥6)	1.19***	(1.08 - 1.31)	1.12*	(1.00 - 1.26)	1.62**	(1.15 - 2.29)
Age	1.01+	(1.00 - 1.02)	0.97***	(0.96 - 0.98)	0.98	(0.95 - 1.01)
Female (ref: male)	1.11*	(1.01 - 1.21)	1.00	(0.90 - 1.12)	0.72*	(0.52 - 1.00)
Education (ref: low)						
- Middle	0.97	(0.89 - 1.07)	1.08	(0.97 - 1.21)	0.85	(0.61 - 1.18)
- High	0.99	(0.89 - 1.11)	1.05	(0.93 - 1.20)	0.82	(0.55 - 1.21)
Marital status (ref: married, living with spouse)						
- Married, living separated	0.96	(0.75 - 1.24)	1.26	(0.96 - 1.66)	1.50	(0.63 - 3.57)
- Single	0.91	(0.73 - 1.13)	0.96	(0.74 - 1.23)	0.99	(0.44 - 2.24)
- Divorced	1.30**	(1.07 - 1.58)	1.05	(0.82 - 1.35)	1.57	(0.77 - 3.19)
- Widowed	1.05	(0.91 - 1.22)	1.07	(0.90 - 1.27)	0.96	(0.55 - 1.66)
Living situation (ref: alone)						
- Living together with spouse/partner/relative	1.02	(0.89 - 1.17)	1.13	(0.96 - 1.33)	0.72	(0.42 - 1.22)
- Retirement home/assisted living/ nursing home	1.10	(0.94 - 1.30)	0.98	(0.80 - 1.21)	0.87	(0.47 - 1.59)
Health insurance (ref: statutory)						
- Private health insurance	1.37***	(1.14 - 1.65)	0.83	(0.65 - 1.05)	1.67	(0.90 - 3.11)
High Social support (ESSI) (ref: low)	1.03	(0.94 - 1.13)	1.10+	(0.99 - 1.22)	1.14	(0.79 - 1.64)
Cognitive function (MMSE)	0.98*	(0.97 - 1.00)	1.00	(0.98 - 1.02)	1.01	(0.95 - 1.08)
Chronic disease score (CDS)	1.05***	(1.03 - 1.06)	1.03**	(1.01 - 1.04)	1.12***	(1.06 - 1.17)
Functional impairment (ADL) (ref: no)	1.17***	(1.08 - 1.26)	1.09*	(1.00 - 1.19)	1.79***	(1.29 - 2.49)
Visual impairment: (ref: no)	0.92*	(0.85 - 1.00)	1.01	(0.93 - 1.11)	1.07	(0.79 - 1.44)
Hearing impairment: (ref: no)	0.97	(0.90 - 1.05)	1.14**	(1.05 - 1.25)	1.27+	(0.96 - 1.68)
Random part	yes		yes		yes	
Observations	1,843		1,846		1,843	
Individuals	1,118		1,119		1,118	

Notes: Notes: Incidence-rate ratios (IRR) and odds-ratios (OR) with 95% confidence intervals (95% CI) are reported. Depression - short version of the Geriatric Depression Scale, which contains 15 items (GDS-SF) (29); Educational level (CASMIN) low, middle, high [33]; Social support - German Version of the ENRICHD Social Support

Inventory - ESSI (35), range 5-25, values lower or equal to 18 indicated low social support (36); Cognitive function - Mini-Mental State Examination (MMSE), from 0 (worst) to 30 (best) (39); Chronic disease score (CDS) - burden of multimorbidity, range 0-12, higher scores indicate higher morbidity (38); Functional impairment - 24-item scale (instrumental) activities of daily living (IADL/ADL). Patients who had difficulty on at least one ADL/IADL were classified as functionally impaired (37). *** p<0.001, ** Notes: p<0.01, * p<0.05

Table S2 Sensitivity analysis – results stratified by sex. Determinants of health care utilisation (GP visits, specialist visits and hospital admission) in late life among community-dwelling individuals. Results of multilevel random intercept negative binominal regression (IRR) and the random-effects logistic regression (OR). AgeMooDe - German “Late-life depression in primary care: needs, health care utilisation and costs”.

	Number of GP visits		Medical specialists – number of visits		Hospital admission	
Independent variables	Female	Male	Female	Male	Female	Male
	IRR (95%CI)	IRR (95%CI)	IRR (95%CI)	IRR (95%CI)	OR (95%CI)	OR (95%CI)
Depressive symptoms (GDS)	1.03** (1.01 - 1.05)	1.03* (1.01 - 1.06)	1.03* (1.00 - 1.05)	1.03+ (1.00 - 1.06)	1.07+ (1.00 - 1.14)	1.09+ (1.00 - 1.19)
Age:	1.00 (0.99 - 1.02)	1.01 (1.00 - 1.03)	0.96*** (0.95 - 0.97)	0.97*** (0.96 - 0.99)	0.98 (0.94 - 1.02)	0.97 (0.92 - 1.03)
Education (CASMIN): (ref: Low)						
- Middle	0.96 (0.86-1.07)	1.01 (0.85-1.19)	0.98 (0.85-1.13)	1.30** (1.08-1.56)	0.84 (0.55-1.28)	0.91 (0.53 - 1.58)
- High	0.97 (0.83-1.14)	1.03 (0.88-1.20)	1.07 (0.88-1.31)	1.06 (0.89-1.26)	1.34 (0.75-2.38)	0.54* (0.31-0.94)
Marital status: (ref: Married, living with spouse)						
- Married, living separated with spouse	1.02 (0.73-1.42)	0.95 (0.65-1.39)	1.10 (0.74-1.64)	1.45* (1.00-2.11)	1.78 (0.51-2.11)	1.27 (0.35-4.69)
- Single	0.94 (0.73-1.22)	0.87 (0.55-1.36)	1.11 (0.82-1.52)	0.61+ (0.36-1.02)	1.18 (0.44-3.19)	0.24 (0.03-2.22)
- Divorced	1.35* (1.06-1.72)	1.18 (0.80-1.75)	1.17 (0.85-1.60)	0.90 (0.57-1.40)	1.61 (0.64-4.07)	2.37 (0.66-8.54)
- Widowed	1.07 (0.89-1.29)	1.05 (0.83-1.33)	1.13 (0.89-1.42)	1.10 (0.86-1.41)	1.06 (0.49-2.28)	0.68 (0.28-1.67)
Living situation: (ref: Alone)						
- Living together with spouse/ partner/relative	1.01 (0.85-1.21)	1.12 (0.89-1.42)	1.19 (0.96-1.47)	1.09 (0.86-1.39)	0.72 (0.36 - 1.47)	0.59 (0.24 - 1.42)
- Retirement home/assisted living/ nursing home	1.03 (0.86-1.25)	1.28 (0.92-1.79)	0.95 (0.74-1.21)	1.10 (0.76 - 1.60)	1.05 (0.53 - 2.09)	0.48 (0.12 - 1.92)
Private Health Insurance: (ref: Statutory)	1.64*** (1.27 - 2.13)	1.15 (0.89 - 1.50)	0.71+ (0.48 - 1.04)	0.93 (0.70 - 1.24)	1.44 (0.52 - 3.98)	1.67 (0.72 - 3.91)
Social Support (ESSI): (ref: Low)						
- High	1.10+ (0.99-1.22)	0.90 (0.76-1.07)	1.06 (0.93-1.21)	1.28* (1.05-1.56)	1.38 (0.87-2.20)	0.72 (0.38-1.36)
Cognitive function (MMSE):	0.99 (0.97-1.01)	0.98+ (0.97 - 1.00)	1.01 (0.98-1.03)	0.99 (0.96 - 1.02)	0.93+ (0.86-1.01)	1.20** (1.06-1.35)

Chronic disease score (CDS):	1.05***<br>(1.03 - 1.06)	1.04***<br>(1.02 - 1.07)	1.02*<br>(1.00-1.04)	1.03*<br>(1.00-1.05)	1.09**<br>(1.02 - 1.17)	1.15**<br>(1.05 - 1.25)
Functional impairment (ADL): (ref: No)	1.19***<br>(1.08-1.32)	1.10<br>(0.97-1.25)	1.05<br>(0.94-1.18)	1.11<br>(0.97 - 1.26)	1.97**<br>(1.22-3.16)	1.54+<br>(0.94-2.51)
Visual impairment: (ref: No)	0.92<br>(0.84-1.02)	0.92<br>(0.80-1.05)	0.96<br>(0.85-1.08)	1.09<br>(0.94-1.25)	0.92<br>(0.62-1.36)	1.30<br>(0.80-2.13)
Hearing impairment: (ref: No)	1.01<br>(0.92-1.11)	0.91<br>(0.81-1.03)	1.24***<br>(1.11-1.40)	1.01<br>(0.89-1.16)	1.36<br>(0.94-1.96)	1.10<br>(0.70-1.74)
Observations	1,158	685	1,159	687	1,155	688
Individuals	698	420	698	421	696	422

Notes: Incidence-rate ratios (IRR) and odds-ratios (OR) with 95% confidence intervals (95% CI) are reported. Depression - short version of the Geriatric Depression Scale, which contains 15 items (GDS-SF) (29); Educational level (CASMIN) low, middle, high [33]; Social support - German Version of the ENRICHD Social Support Inventory - ESSI (35), range 5-25, values lower or equal to 18 indicated low social support (36); Cognitive function - Mini-Mental State Examination (MMSE), from 0 (worst) to 30 (best) (39); Chronic disease score (CDS) - burden of multimorbidity, range 0-12, higher scores indicate higher morbidity (38); Functional impairment - 24-item scale (instrumental) activities of daily living (IADL/ADL). Patients who had difficulty on at least one ADL/IADL were classified as functionally impaired (37). *** p<0.001, ** Notes: p<0.01, * p<0.05
